# A Fetal Well-Being Diagnostic Method Based on Cardiotocographic Morphological Pattern Utilizing Autoencoder and Recursive Feature Elimination

**DOI:** 10.3390/diagnostics13111931

**Published:** 2023-06-01

**Authors:** Haad Akmal, Fırat Hardalaç, Kubilay Ayturan

**Affiliations:** Department of Electrical and Electronics Engineering, Gazi University, Ankara 06570, Turkey; firat@gazi.edu.tr (F.H.); kubilay.ayturan@gazi.edu.tr (K.A.)

**Keywords:** diagnostics, cardiotocography, fetal heart rate, fetal well-being, machine learning, classification, feature extraction, feature selection, Bayesian optimization

## Abstract

Cardiotocography (CTG), which measures the fetal heart rate (FHR) and maternal uterine contractions (UC) simultaneously, is used for monitoring fetal well-being during delivery or antenatally at the third trimester. Baseline FHR and its response to uterine contractions can be used to diagnose fetal distress, which may necessitate therapeutic intervention. In this study, a machine learning model based on feature extraction (autoencoder), feature selection (recursive feature elimination), and Bayesian optimization, was proposed to diagnose and classify the different conditions of fetuses (Normal, Suspect, Pathologic) along with the CTG morphological patterns. The model was evaluated on a publicly available CTG dataset. This research also addressed the imbalance nature of the CTG dataset. The proposed model has a potential application as a decision support tool to manage pregnancies. The proposed model resulted in good performance analysis metrics. Using this model with Random Forest resulted in a model accuracy of 96.62% for fetal status classification and 94.96% for CTG morphological pattern classification. In rational terms, the model was able to accurately predict 98% Suspect cases and 98.6% Pathologic cases in the dataset. The combination of predicting and classifying fetal status as well as the CTG morphological patterns shows potential in monitoring high-risk pregnancies.

## 1. Introduction

Cardiotocography (CTG) is a non-stress diagnostic method for monitoring the fetal well-being during the third trimester or during labor [1]. CTG continuously records maternal uterine contractions (UC) via a pressure transducer placed on the abdominal wall, and fetal heart beats (FHR) via an external ultra-sound probe on the maternal abdominal wall. The simultaneous readouts can be displayed in real time. Based on expert criteria [1], CTG is typically interpreted by clinicians as Normal, Suspect or Pathologic. In developed countries, CTG is one of the most popular choices of assessing the fetal well-being [2]. Some authors are even arguing that CTG is being overused in low-risk cases [2]. There is a connection between CTG and perinatal mortality and morbidity, as a pathological CTG result is linked to a low APGAR score and neonatal intensive care units (NICU) [3]. The status of fetus can also be used to observe fetal distress. Depending on the underlying causes, the degree of the distress, and the promptness of medical interventions, fetal distress can result in a variety of outcomes. If fetal distress is temporary, then it can be resolved by changing the mother’s position, administering oxygen (to the mother), adjusting intravenous fluids, or performing an emergency cesarean section (around the end of third trimester), if necessary. All these steps can help improve the baby’s condition and lead to a positive outcome. However, if fetal distress is prolonged, then it can lead to long-term negative outcomes such as cognitive impairments, learning disabilities, motor impairments, conditions such as cerebral palsy or even childbirth (in rare cases). Lack of oxygen usually leads to prolonged fetal distress [4]. In some cases, it also results in birth asphyxia (which accounts for approximately 900,000 neonatal deaths annually) [5]. Fetal mortality is more common in low-income nations than in high-income nations overall, underscoring the differences in healthcare access and resources across these areas. Although the global neonatal mortality rate (per 1000 live births) has decreased from 36.7 (1990) to 17 (2020) in the past three decades, it is still comparatively higher for low-income regions [6]. Even in high-income regions, one of the most common causes of fetal death was complications of the placenta (which is related to fetal distress too). In the District of Columbia, USA, 24.4% fetal deaths (in 2020) were due to complications of the placenta [7]. Hence, recognizing the status of the fetus is important in assessing the fetal well-being. CTG can provide early indication of fetal distress. CTG tests are time- and resource-efficient; thus, they mitigate patient discomfort especially if numbers are high. CTG tracing patterns such as fixed FHR baselines, loss of FHR variability, and absence of accelerations, are indicative of a non-reassuring case [8,9]. CTG is visually interpreted by an expert and to supplement this activity, automated mechanisms are being proposed. Machine learning can be used to detect fetal hypoxia and status of the fetus [10,11,12,13]. This research proposes a diagnostic model that classifies and predicts the fetus status as well as the CTG morphological patterns. Missing data in CTG recordings can have a significant impact on the interpretation of the fetal well-being and can lead to suboptimal decision-making in managing labor and delivery. Missing data can lead to the following issues:

- An incomplete assessment of the fetal well-being because the CTG recording contains crucial information regarding the fetal heart rate and uterine contractions. As a result, chances to detect fetal distress or hypoxia early may be lost.
- Misinterpretation of the CTG pattern. As a result, unneeded interventions such as emergency cesarean sections may occur when they were not needed.
- A delay in the decision-making and the proper management of labor and delivery. As a result, this may have negative effects on the well-being of the mother and the fetus.

Issues such as missing values can be resolved during the preprocessing stage. Thus, preprocessing of the CTG dataset is quite necessary. In [14], an algorithm is described that involves two iterative steps for filling in missing data. In the first “reconstruction step”, an adaptive dictionary is used to reconstruct the signal that leads to estimation of missing data, and then, in the second step, a new dictionary is calculated using the KSVD (k-singular value decomposition) algorithm based on the reconstructed signal from the first step. These two steps are repeated until convergence is achieved. The algorithm displayed good results particularly for consecutive missing samples. The dataset [15] considered for this research was the result of an automated analysis of the SisPorto 2.0 program [16]. The proposed program solved the missing data problem. The hypothesis for this research is that by using a machine learning model based on feature extraction, feature selection, and Bayesian optimization, it is possible to accurately diagnose and classify the various fetal conditions (Normal, Suspect, Pathologic), as well as the CTG morphological patterns, offering a potential decision support tool for managing pregnancies. Elaborating the hypothesis, the following objectives are proposed for this research: to diagnose the fetal well-being, the proposed objectives of this study are to counter the imbalanced nature of the CTG dataset; to propose an encoder-bottleneck information variable (discussed in Methodology section); to implement feature extraction (to counter the comparatively larger size of the CTG dataset achieved after implementing the first objective); to implement feature selection; to perform Bayesian optimization (to further increase the performance of the proposed model); to implement classification and to formulate a method to integrate all the above-mentioned modules.

## 2. Related Work

Several comparative studies [17,18,19,20,21,22,23,24,25] have been conducted to evaluate the performance of various classifiers on the CTG dataset [15]. These studies have utilized a variety of classifiers such as Artificial Neural Network (ANN), Long Short-Term Memory (LSTM), eXtreme Gradient Boosting (XGB), Support Vector Machine (SVM), K-Nearest Neighbor (KNN), Light Gradient Boosting Machine (LGBM), Random Forest (RF), Ada Boost, Bagging and Stacking, Decision Tree (DT), Naïve Bayes (NB), Logistic Regression (LR), Classification and Regression Trees (CART), Levenberg–Marquardt (LM) backpropagation, Resilient Backpropagation (RP), and Gradient Boosting Machine (GBM). The studies achieved accuracy rates ranging from 83.65% to 96.61%. The studies have generally concluded that RF is the best-performing classifier. The NB classifier combined with the Firefly algorithm and random feature selection resulted in an accuracy of 86.54% (8 features) [26]. A stacked model approach was used in [27], which included a combination of multiple models, to counter the imbalance in the CTG dataset [15] with its anti-interference traits. The results showed an accuracy of 96.08%. An AutoML approach with Synthetic Minority Oversampling Technique (SMOTE) was implemented [28] for the CTG dataset [15]. Out of all the models used in PyCaret, LGBM had an accuracy of 95.61%. Authors in [29] proposed their own model (95% accuracy) for feature selection after implementing SMOTE on the imbalanced CTG dataset [15]. The Differential Privacy (DP) framework-based neural network model (91% accuracy) [30] had two binary classifiers that classified the CTG dataset [15]. An a priori algorithm-based classification model was proposed in [31]. The proposed model (with Adaboost and RF) had feature selection as well. In addition, the suspect class of the CTG dataset [15] was split into normal and pathological classes to increase overall model accuracy. Relevant CTG features of the CTG dataset [15] were selected via Principal Component Analysis (PCA) and then fed to an SVM-AdaBoost model (93% accuracy [32]). The adjustment parameters were tweaked via a self-learning algorithm in a Fuzzy C means clustering-based ANFIS model [33], and model accuracy was 96.39% when 9 features were manually selected from the CTG dataset [15]. In [34], it was observed that the two outputs (of the CTG dataset [15]) have shared representations which allowed the model to utilize shared features between the two outputs.

The inspiration of using different modules (discussed in Section 3.6) came from the above-mentioned related literature. Hence, the proposed model of this study includes modules such as a method for balancing the dataset, feature extraction, feature selection, and hyperparameter optimization. The main difference between the proposed model and the above-mentioned related literature is that not all the modules used in the proposed model are utilized together in such a manner. The type of method for balancing the dataset, feature extraction, feature selection, hyperparameter optimization method, and classification mechanism was selected based on their respected performances in the related literature review. A method for balancing the dataset was implemented using SMOTE (Appendix A.4), feature extraction was implemented using Autoencoder (Section 3.1), feature selection was implemented using Recursive Feature Elimination (Section 3.2), hyperparameter optimization was implemented using Bayesian optimization (Appendix A.1), and classification was implemented using Random Forest (Section 3.3).

## 3. Materials and Methods

### 3.1. Feature Extraction

Feature extraction (FE) is a term used for all those techniques that allow new features to be derived from an existing dataset. These new features could be then used again to obtain the original dataset. Based on simplicity and flexibility, autoencoder (AE) was selected for this research. An autoencoder first tries to learn the patterns, and relationship between the features of an input data, then tries to recreate the original input. An autoencoder balances the following two traits:

- Sensitive enough to inputs in such a manner that it can accurately build a reconstruction.
- Insensitive enough to inputs in a such a manner that it does not simply memorize (overfit) the input training data.

This balance restricts the autoencoder to learn only those variations in the input that allows it to recreate the original input, while ignoring the redundant entries in the input. A loss function (Equation (1)) has a reconstruction loss (RL) that encourages the autoencoder to be sensitive to input and a regulator that discourages full memorization of the input by the autoencoder.

(1) $$LossFunction=RL(x,x^{\prime })+Regulator$$

A typical autoencoder has three parts: The *encoder* is a set of multiple layers that gradually compresses the input data that can be fed to a bottleneck. The encoder can be composed of multilayered perceptron (MLP) or convolutional blocks. The purpose of the *bottleneck* is to limit the neural network to not memorize the whole input data, but to memorize only the trends in the input entries. The *decoder* is the opposite of an encoder.

### 3.2. Feature Selection

Feature selection (FS) is the process in which the dimension of a dataset can be reduced and, in some cases, the efficiency of the model can also be increased if the least important or redundant features of a dataset are removed. In scenarios where a large amount of system memory is used due to a large dataset, feature selection is quite useful. Due to its flexibility and robustness, Recursive Feature Elimination (RFE), a wrapper-type supervised method, was used in the proposed model for feature selection. RFE eliminates the least important feature one by one until a desired stage is reached. The RFE algorithm is as follows:

1. Train the classifier.
2. Compute the ranking criterion for all the features.
3. Remove the feature with the smallest-ranking criterion and repeat till a stopping criterion is achieved.

### 3.3. Classification

In machine learning, classification is an arrangement of entries in different groups based on an established criterion. Random forest is selected for this research based on the literature review. RF is an assemblage learning method for performing regression as well as classification. It can construct multiple decision trees during the training of the model and then merge them to obtain a more accurate result. It counters the issues of over-fitting by decision trees. At each node, the decision to split classes is achieved via “Information Gain”. There are many methods to achieve this information gain. One method used in this research is entropy (defined as Equation (2)).

(2) $$E=\sum_{i=1}^{U}-f_{i}log\left(f_{i}\right)$$

Here, $f_{i}$ is the frequency of label *i* at a node and *U* is the number of unique labels. Then, the information gain is given as Equation (3).

(3) Gain(V,X)=E(V)−E(V,X)

Here, *V* is the target variable, *X* is the feature where a split will occur, and Entropy *E(V, X)* is the entropy calculated after the data are split on feature *X*. Another approach to find the split criterion is by using the Gini index (or impurity). This is a measure of inequality (impurity) of a set of values. It does so by measuring the probability for a random instance being misclassified when chosen randomly. This is in turn used to assess the quality of a split. If $p_{i}$ is the proportion of values in the set that belongs to class *i* and *c* represents the number of classes in the target variable, then *Gini impurity* is given as Equation (4).

(4) $$Gini\;impurity=1-\sum_{i=1}^{c}{\left(p_{i}\right)}^{2}$$

### 3.4. Performance Analysis

Analyses of the proposed model were evaluated using precision, recall, F1-score, accuracy, and specificity metrics (Table 1). The proposed model was cross-validated using 5-fold stratified cross-validation. The reason for using stratified cross-validation was that the CTG dataset [15] was imbalanced. Even though the imbalance was resolved via SMOTE (as discussed in Section 3.5), still precaution was taken to avoid the inherent imbalance nature of the dataset. Receiver operating characteristic (ROC), area under the ROC curve (AUC), and Precision-Recall curve were also calculated. The reason for utilizing ROC and AUC in this study was due to the fact that these performance metrics are used to convey the feasibility of using this proposed model to the readers, to encourage them to implement this model in their studies as well. Another reason for using ROC and AUC in this study was to provide an intuitive interpretation of the results of the proposed mode. Thus, if this article is read by non-ML (machine learning) background readers, even then they can easily understand the performance of the proposed model. Since the area of this article also includes the medical domain, medical experts could understand the performance of the proposed model as well. To further increase the interpretability of the proposed model, the SHAP (SHapley Additive exPlanations) (discussed in Appendix A.3) analysis was also performed.

### 3.5. Dataset Overview

The dataset used in this study was acquired from [15], which is the result of the Omniview-SisPorto 2.0 program [16]. Since the SisPorto 2.0 program performed an automated analysis of cardiotocograms (for both ante- and intrapartum tracings) that closely followed the International Federation of Gynecology and Obstetrics (FIGO) guidelines. The resulting dataset also conformed to the FIGO criteria. The dataset contains 21 features (Table 2) from 2126 CTG recordings grouped into three classes (Normal, Suspect, and Pathologic) and ten diagnostic classes (Table 3). All features and classes had been verified by 3 experts. The fetal condition was assessed by the criteria in Table 4.

**Figure 1:**
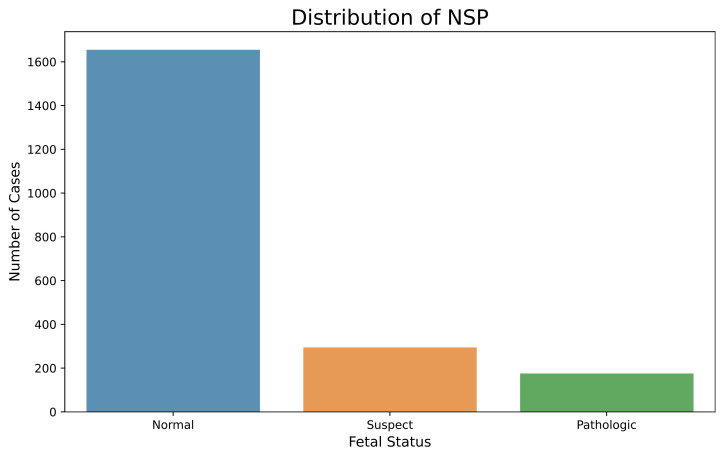
Imbalanced CTG Dataset for Fetal Status.

**Figure 2:**
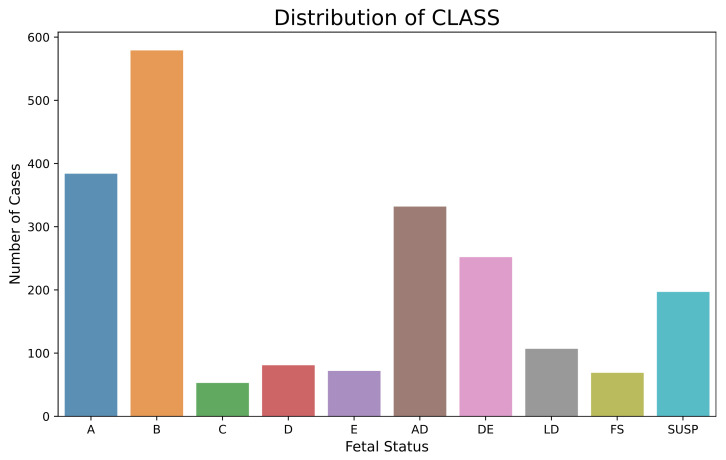
Imbalanced CTG Dataset for Morphological Pattern.

The imbalanced nature of the CTG dataset, as observed from Figure 1 and Figure 2, was countered by implementing SMOTE [35]. SMOTE generates synthetic entries in the dataset by interpolating minority class entries into the feature space of data. The new instances are placed between a minority sample and its k neighbors. The working principle of SMOTE is given in Appendix A.4.

Correlation between variables indicates a dependence between them. Correlation does not directly affect a classification model; however, the presence of a significant number of correlated variables in a dataset can indirectly affect the model by feeding redundant variables (features) to the model that can decrease the quality of the classification model. If ρ is the Spearman’s rank correlation coefficient, $d_{i}^{2}$ is the square of the difference in the ranks of two variables for each pair, and *n* is the number of pairs, then the Spearman correlation formula is given in Equation (5).

(5) $$\rho =1-\frac{6\sum d_{i}^{2}}{n(n^{2}-1)}$$

**Figure 3:**
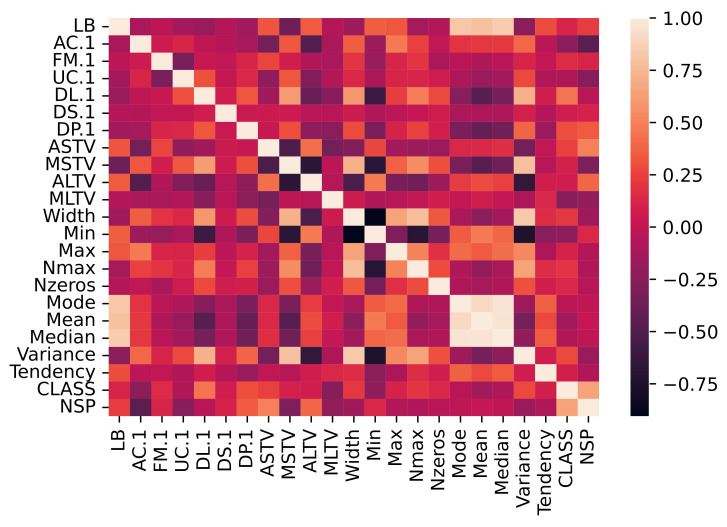
Spearman Correlation of the CTG Dataset.

It was observed from the Spearman correlation heatmap (Figure 3) that there was a strong (>0.7) correlation between FHR Baseline (LB) and FHR histogram descriptors (Mode, Mean, and Median), between Width, Minimum, Maximum of FHR histogram, and number of histogram peaks. Some of the FHR histogram descriptors (Mode, Mean, and Median) themselves were correlated to each other. This was also corroborated by [36], where the author also used Correlation Analysis (CA) to identify the relationship between the FHR histograms and Explanatory Data Analysis (EDA) to identify the relationship between accelerations and decelerations of uterine contractions. The similarities can negatively affect the quality of the model prediction. This provided another reason to implement a feature extraction technique to reduce the number of redundant features. The issue due to strongly (positive and negative) correlated redundant features will be resolved in the simulation part.

The CTG dataset was preprocessed by deletion of multiple empty rows and a few empty columns that contained no information. Furthermore, the predictor columns in the dataset have no Not a Number (NaN) entry. In addition to the features in Table 2, there was another feature DR (Repetitive decelerations) in the CTG dataset [15]; however, it was removed because the authors [16] performed a chi-squared test on all the features of the dataset and found that p(K-W) = 1 for DR. This removal was also corroborated in [31]. For fetal status, the target output was NSP (with 3 classes), and for CTG morphological pattern, the target output was CLASS (with 10 classes). Dataset standardization was performed using “z-score”. If μ is the mean of the sample and σ is the standard deviation of the sample, then the formula used for the z-score is given in Equation (6).

(6) $$z=\frac{x-\mu }{\sigma }$$

### 3.6. Methodology

**Figure 4:**
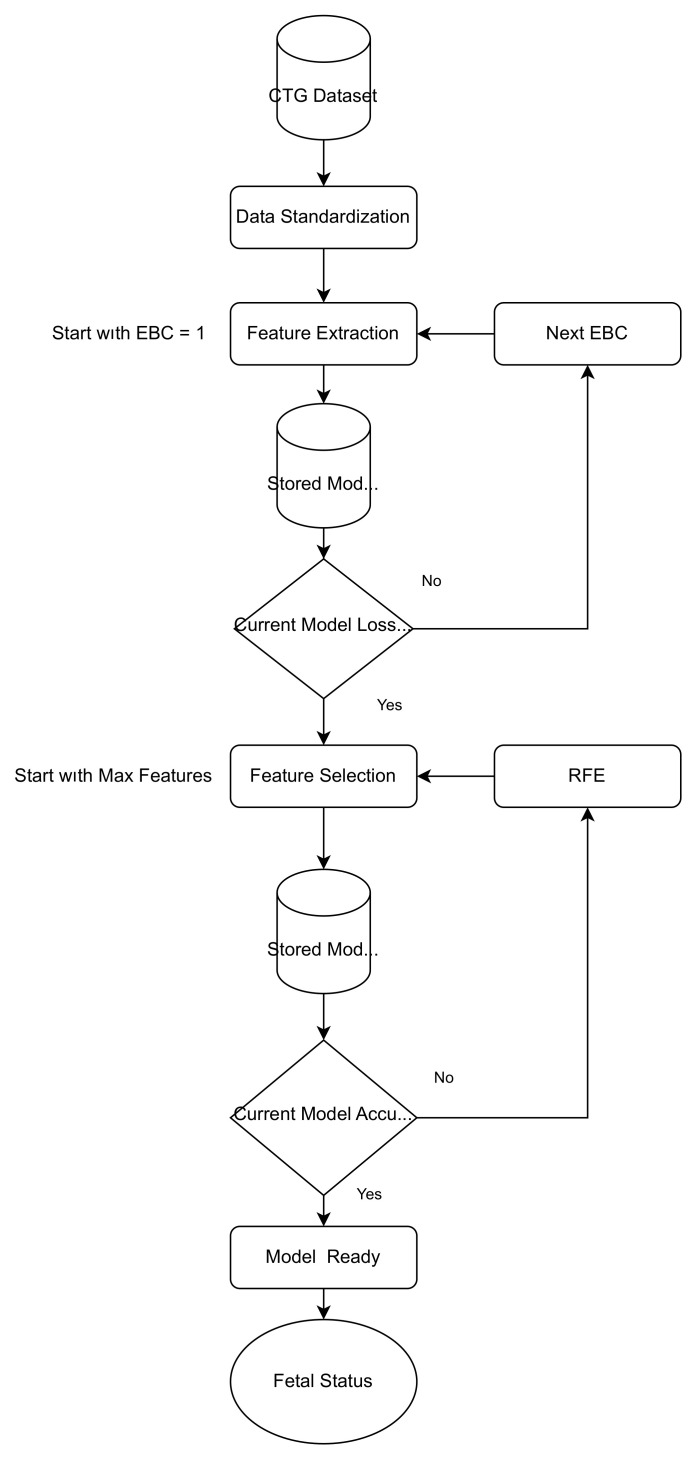
Flowchart of the Proposed Algorithm.

This study adheres to the STROBE guidelines [37] for reporting observational studies. After implementing SMOTE on the CTG dataset, the size of the input matrix was increased to 4965. For this comparatively larger dataset, a model was proposed that was inspired from Feature Extraction (via autoencoders) and Feature Selection (via RFE). Both feature selection and feature extraction modify the input matrix; the main difference is that feature selection keeps the original input matrix intact and removes the features based on their ranks, whereas feature extraction creates new features while automatically removing the undesirable features. The proposed model aimed to reduce the redundant entries and dimensionality of the dataset. The flowchart of the proposed algorithm is shown in Figure 4.

The primary task of the FE module was to find the encoder bottleneck information (EBI). The number of neurons (EBN: encoder bottleneck neurons) in the bottleneck layer can be found in Equation (7).

(7) $$EBN=\frac{OF\_max}{EB_{C}}$$

OF_max is the maximum number of original features, and $EB_{C}$ is the proposed encoder bottleneck coefficient whose value would change from 1 to 2, depending on the outcome of the algorithm. The initial value of $EB_{C}$ was set as 1. Results showed that $EB_{C}$ > 2 resulted in low-quality data reconstructions. Traditionally selecting the dimensions of EBI is an ad hoc and non-standard process, but the results of the proposed model showed that $EB_{C}$ = 1.5 resulted in a good compromise between dimensionality reduction and keeping the minimum number of features for better classification. The secondary task of the FE module was to reduce the dimensions of the dataset. The new dataset had new features that represented the original dataset. In the FS module, RF was used as an estimator for the RFE. The advantage of using RFE here was that it was unconcerned with the type of input features, as it relied on the feature importance. The final model, with the help of Random Forest, was then used to classify the fetal status and CTG morphological pattern. The final selected model was also fed to a Bayesian optimization (BO) module. BO sequentially pursues the global optimum with the least number of iterations, while treating every problem as a black box. It seeks a balance between exploration (collect more information), and exploitation (finalizing the best decision on information) [38]. For RF, the hyperparameters such as split criterion (the function to measure the quality of a split), class weights, maximum number of features for a split and number of trees in a forest (estimators) were fine-tuned to find the optimal solution. The CTG dataset was annotated by SisPorto 2.0 [16]; thus, the predictors/features had a form of feature extraction. However, from Figure 4, it can be observed that the second feature extraction was performed after the implementation of SMOTE (that was used to counter the class imbalance nature of the CTG dataset). The need for another feature extraction originated from the fact that now the dataset was comparatively larger; hence, there was a possibility that there was some redundant information in the new dataset and the larger dataset would increase the computational costs.

## 4. Results

The simulations were performed in a Python 3.8 environment. The simulations were divided into two parts. Part 1 covers the fetal status aspect of the CTG dataset, whereas Part 2 covers the CTG morphological pattern aspect of the CTG dataset. For comparison purposes, RF (without the proposed algorithm) was also used on the CTG dataset. For both parts, the training to testing ratio was set as 75:25. RF can naturally support multiclass classification, so it was directly used for this multiclass dataset.

### 4.1. Fetal Status Classification

The performance analysis (using the performance metrics given in Table 1) of the proposed model for the fetal status is given in Table 5. For an easier comparison, the table also contains entries from the case in which only basic RF (without the proposed algorithm) was used.

The model accuracy of the proposed model for CTG fetal status was 96.62% (with 13 features). Whereas if only basic RF was used on the same dataset (with all 21 features), an accuracy of 93.61% was achieved. The confusion matrix of the proposed model for fetal status is shown in Table 6. For ease of comparison, the entries in the confusion matrix are depicted as percentages and the table also contains entries from the case in which only basic RF (without the proposed algorithm) was used.

**Figure 5:**
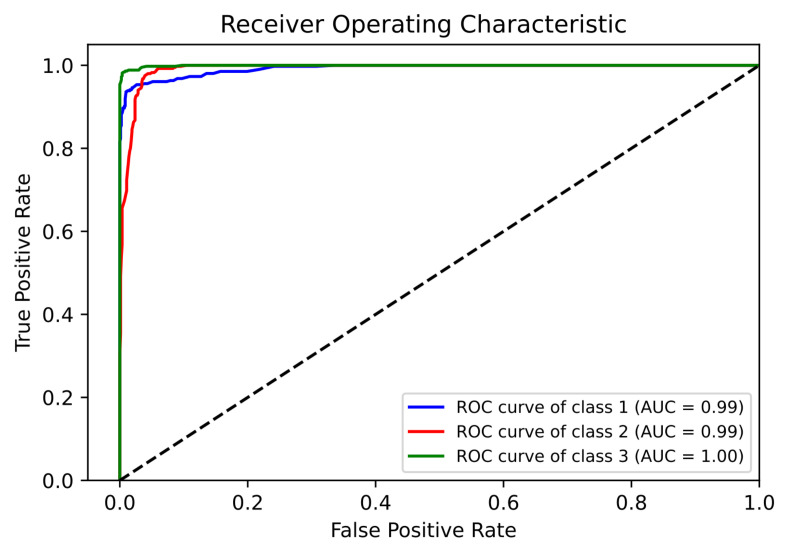
ROC Curve and AUC of the Proposed Model for Fetal Status.

**Figure 6:**
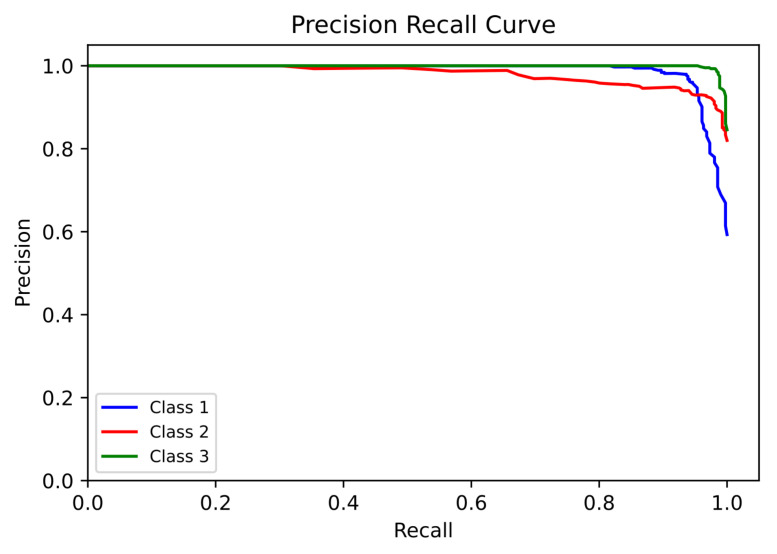
PR Curve of the Proposed Model for Fetal Status.

The ROC (with AUC) and PR were measured for all three classes (Class 1 = Normal, Class 2 = Suspect, and Class 3 = Pathologic) individually, as observed in Figure 5 and Figure 6, respectively.

**Figure 7:**
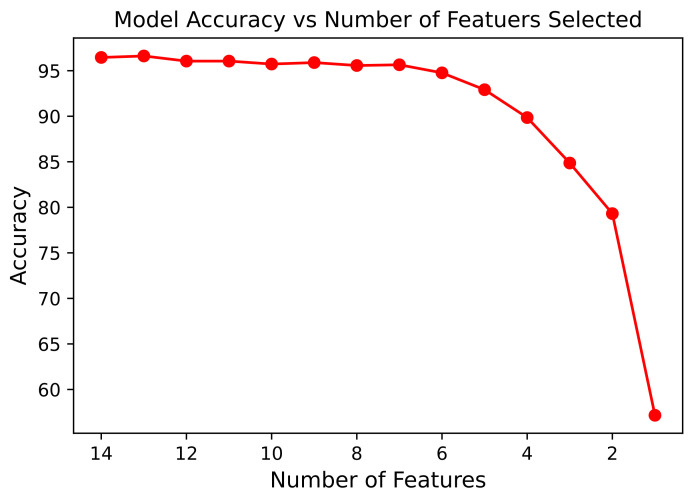
Relation of Model Accuracy with the Number of Features Selected during a Complete Run for Fetal Status.

The variation in the model accuracy during the full run of the proposed model for fetal status can be observed in Figure 7. The highest accuracy, 96.62%, was achieved by the proposed model, when 13 features were selected.

### 4.2. CTG Morphological Pattern Classification

The performance analysis (using the performance metrics given in Table 1) of the proposed model for the CTG morphological pattern is given in Table 7. For an easier comparison, the table also contains entries from the case in which only basic RF (without the proposed algorithm) was used.

The model accuracy of the proposed model for the CTG morphological pattern was 94.96% (with 14 features). Whereas if only basic RF was used on the same dataset (with all 21 features), an accuracy of 87.22% was achieved. The confusion matrix of the proposed model for the CTG morphological pattern is shown in Table 8. For ease of comparison, the entries in the confusion matrix are depicted as percentages, and the table also contains entries from the case in which only basic RF (without the proposed algorithm) was used.

**Figure 8:**
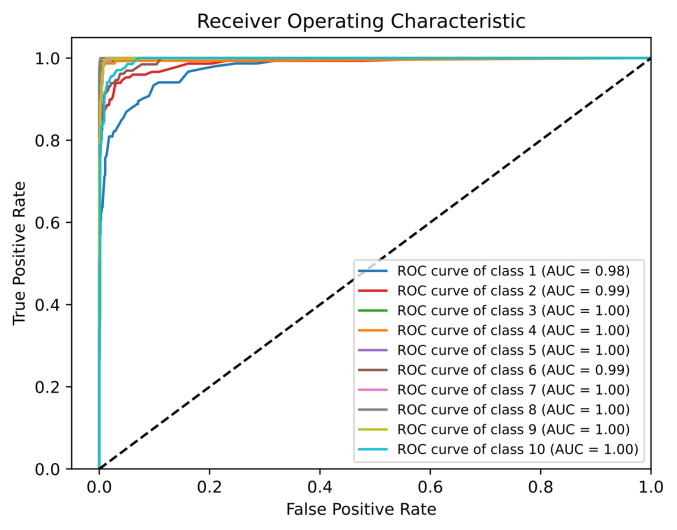
ROC Curve and AUC of the Proposed Model for CTG Morphological Pattern.

**Figure 9:**
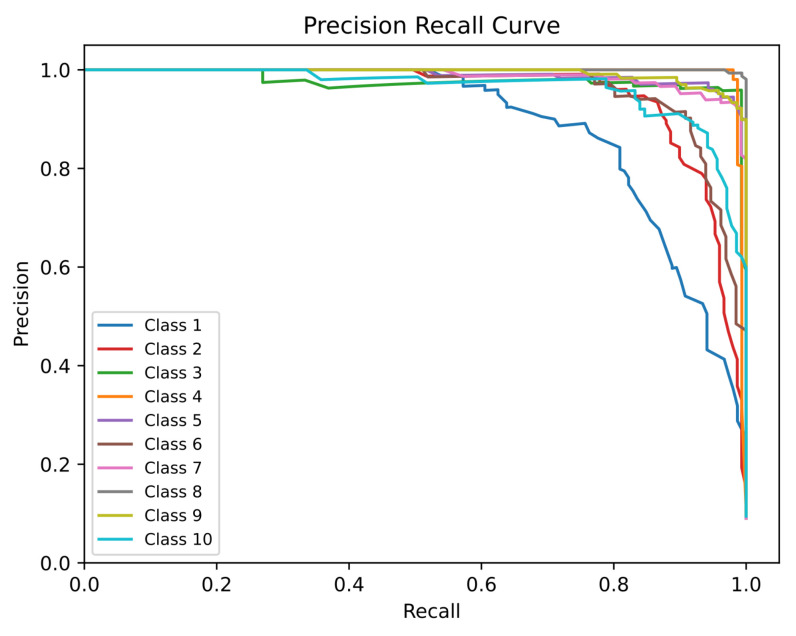
PR Curve of the Proposed Model for CTG Morphological Pattern.

The ROC (with AUC) and PR were measured for all ten classes (Class 1 = A, Class 2 = B, Class 3 = C, Class 4 = D, Class 5 = E, Class 6 = AD, Class 7 = DE, Class 8 = LD, Class 9 = FS, and Class 10 = SUSP) individually, as observed in Figure 8 and Figure 9, respectively.

**Figure 10:**
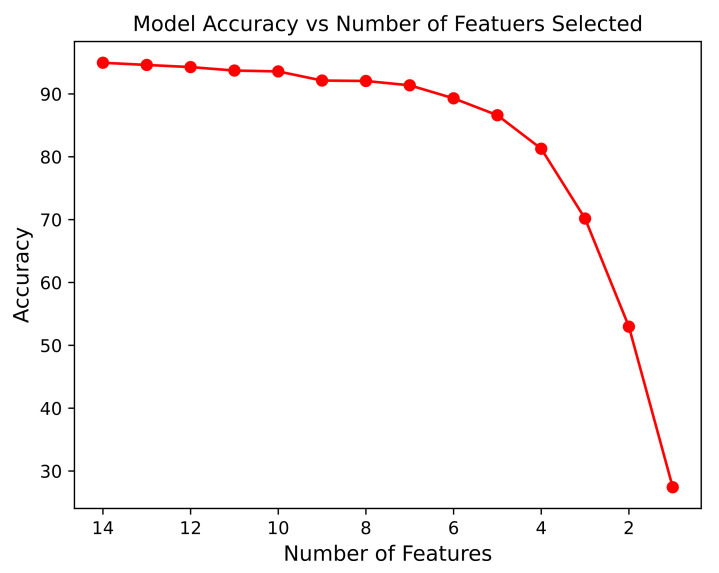
Relation of Model Accuracy with the Number of Features Selected during a Complete Run for CTG Morphological Pattern.

The variation in the model accuracy during the full run of the proposed model for the CTG morphological pattern can be observed in Figure 10. The highest accuracy, 94.96%, was achieved by the proposed model, when 14 features were selected.

Figure 7 displays the complete run for fetal status (in which 3 conditions of the fetus were used as target output), whereas Figure 10 displays the complete run for the CTG morphological Pattern (in which 10 CTG classes were used as target output). The difference between the accuracy of both graphs stems from the fact that for the fetal status case, the target output had only three classes; thus, it was easier to classify that model. Whereas for the CTG morphological pattern model, the target output had 10 classes (refer to the dataset subsection) and it was comparatively difficult to obtain a better classification. Still, the proposed model presented good results for the latter case as compared to using only the basic RF classifier.

### 4.3. Overview of Bayesian Optimization

The main reasons for using Bayesian optimization in this proposed study are to efficiently explore the hyperparameter space, to reduce computational cost in fine-tuning the hyperparameters, and to improve the overall performance of the proposed model. For instance, for the fetal status part, if 13 features were selected (after the RFE module) and no Bayesian optimization was used, then the accuracy would be 96.54%. However, if Bayesian optimization is used after the RFE module, then the accuracy for 13 features is 96.62%. In essence, Bayesian optimization fine-tunes the proposed model and yields better results. Performance metrics table and confusion matrix of both the above-mentioned cases are given in Appendix A.2 for comparison. The optimum hyperparameters of the proposed model for fetal status and for the CTG morphological pattern obtained after the Bayesian optimization module are given in Table 9.

### 4.4. SHAP Analysis

The SHAP summary plot is Beeswarm-type plot, in which the features are represented in the y-axis (the features are sorted with respect to their importance) and the SHAP values are represented in the x-axis (the SHAP measures the contribution of each feature to predicted output). The SHAP output was different for both cases, as for fetus status, the target output consisted of 3 classes, whereas for the CTG morphological pattern, the target output consisted of 10 classes. After the implementation of autoencoder, the new features were labeled as New Extracted Features (NEFs), which ranged from NEF 1 to NEF 14. The low feature value was depicted as a blue dot, whereas a high feature value was depicted as a red dot. For non-binary cases (e.g., in this research), the color range was depicted between blue and red, with purple being the middle feature value. The dots represented individual SHAP values for each data point in the test set. The horizontal bars, along the x-axis, represented the range of the SHAP values for each feature, whereas the length of those bars depicted the extent of the effect each feature has on the model.

**Figure 11:**
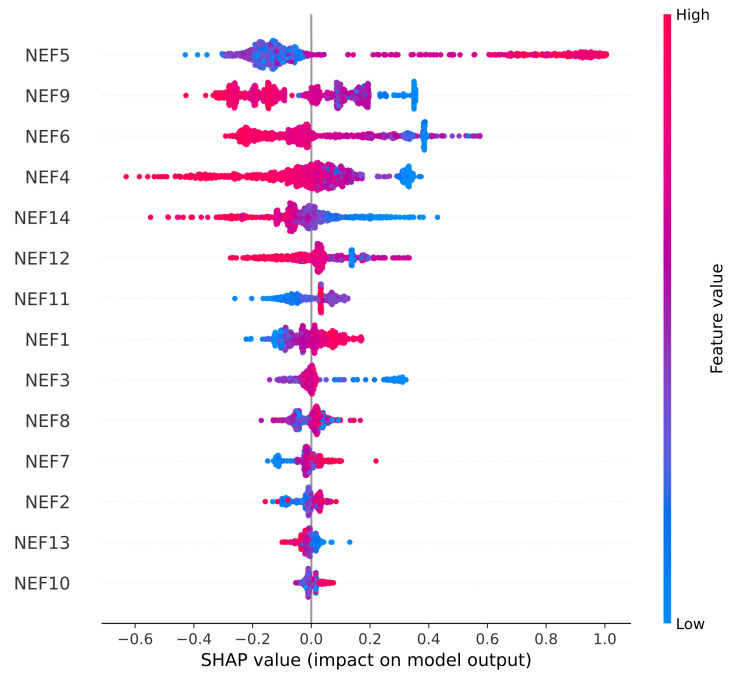
SHAP Analysis Summary Plot for Fetal Status.

For the case of fetal status, in Figure 11, it can be observed that NEF5 has the highest positive impact on the model. NEF4 has the highest negative impact on the model. However, it should be noted that NEF9, NEF6, NEF4, and NEF14 have a high negative impact on the model.

**Figure 12:**
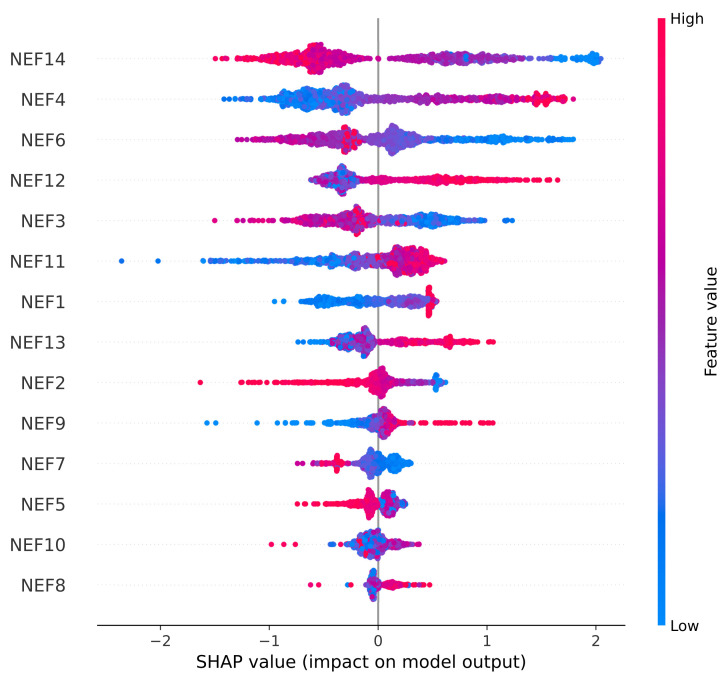
SHAP Analysis Summary Plot for CTG Morphological Pattern.

For the case of the CTG morphological pattern, in Figure 12, it can be observed that NEF14 has the greatest positive impact on the model; however, the strength of the impact lies between low to medium (as observed by the blue and purple colored dots, respectively). NEF11 has the greatest negative impact on the model. Moreover, NEF4, NEF6, and NEF12 have a high positive impact on the model.

The main difference between the two graphs is that for the fetal status part, the NEFs had a high, positive and negative (maximum impact reached around 1.0) impact on the model and also comparatively less features had a significant impact on the model. Whereas for the CTG morphological pattern part, the NEFs had a comparatively higher impact (maximum impact reached around 2.0) on the model and also more features had a significant impact of the model.

In [39], when SHAP was implemented, there were some original features (such as NZEROS: Number of Histogram Zeros, and DS: severe decelerations) that had no impact on the model whatsoever. In this proposed model, all those irrelevant features had been removed via the proposed algorithm. Thus, all new features had an impact on the model output.

## 5. Discussion

The general trend in the relationship between number of features and the accuracy of the proposed model has a negative relation, with fewer number of features leading to lower model accuracy (as observed in Figure 7 and Figure 10).

The performance analysis metrics (Table 5) of the proposed model ranged from 0.92 to 0.99. This is a significant improvement from when only RF was used on the CTG dataset. When basic RF was used (without the proposed algorithm), the Precision and Recall values of the suspect case were very low (0.83 and 0.69, respectively), whereas in the proposed model, those values were 0.92 and 0.98, respectively. For the confusion matrix (Table 6) of the proposed model, a great reduction was achieved in the “incorrect” predictions of suspect and pathological cases. When basic RF was used (without the proposed algorithm), the suspect cases that were incorrectly predicted as normal cases were 26.4%, whereas using the proposed model, this incorrect prediction fell to only 1.5%, a decrease of 94.31% in the incorrect predictions between normal and suspect cases. For a sensitive field such as fetal well-being, the reduction in incorrect prediction is a good aspect of this proposed model. The ROC (Figure 5) and PR (Figure 6) curves for the fetal status case provide good insight about the ability of the proposed model to accurately predict all the three classes with good confidence (as all AUC values are above 0.99). The most important conclusion from the ROC and PR curves is that the model works very good in classifying and predicting the pathological cases. In the medical context, the pathological cases are more concerning than the normal cases. This is because pathological cases need immediate care (as observed from Table 4), so that the well-being of the fetus can be corrected. Although the basic RF classifier (without the proposed algorithm) was able to display good results for the normal cases, the suspect and pathological cases were not being predicted with good confidence level. Many suspect cases were incorrectly predicted either as Normal or Pathologic. Considering the medical implications, this incorrect prediction poses more harm compared to a normal case being incorrectly predicted as either suspect or pathological. In the case of fetal status classification, the accurate classification of pathological and suspect cases holds more significance than the classification of normal cases. Thus, the proposed model was able to increase the confidence levels for predicting both suspect and pathological cases.

The model accuracy of the proposed model for the CTG morphological pattern case was 94.96%. This was an increase of 8.87% as the accuracy was 87.22% when only RF was used without the proposed algorithm. For the basic RF classifier, only A, B, and LD had comparatively better predictions, whereas there were significant incorrect predictions for the rest of the classes in the CTG morphological pattern case, as observed in Table 7. Moreover, for the class E (shift pattern between Calm Sleep, CLASS A and Suspect pattern, CLASS SUSP), the correct predictions, while using only basic the RF classifier, were only 45.8% (with a recall value of 0.45). However, in the proposed model, the incorrect predictions were significantly reduced throughout all the classes. Another improvement was observed in the F1-score, where all morphological patterns displayed good metrics. All classes (except class A) had an F1-score of above 0.91. The good performance of the proposed model can also be highlighted via a confusion matrix (Table 8). For instance, for class E, the correct prediction increased to 98.7%, a percentage increase factor of 115.5%. In addition, the recall value of class E increased to 0.98. Moreover, the important pathological and suspect-related classes (such as FS and SUSP) have comparatively lesser incorrect predictions in the proposed model as compared to using only the basic RF classifier. The average correct predictions of the CTG morphological pattern using the basic RF classifier (without the proposed algorithm) was 80.94%. Whereas using the proposed model, the average correct predictions across all classes were 94.99%.

**Figure 13:**
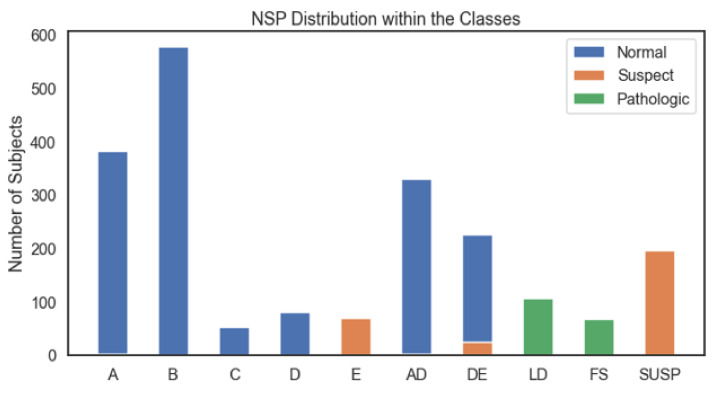
NSP Distribution within the CTG Morphological Pattern CLASS.

Before discussing the ROC and PR curves of the proposed model for CTG morphological pattern, the relationship between fetal status types and the CTG morphological pattern classes should be discussed. As observed from Figure 13, the fetal status classes are distributed over the whole CTG morphological pattern classes. Classes A, B, C, D equate to the normal fetal case. Classes AD and DE equate to the mostly normal fetal case with a minority of suspect case. Classes SUSP and E equate to the suspect case. Class E has a shifting pattern that shifts between a normal calm sleep and a suspected pattern. Moreover, classes LD and FS equate to the pathological case. Although the ROC (Figure 8) and PR (Figure 9) curves of the proposed model for CTG morphological pattern are better than basic RF (without the proposed algorithm), only class A has a decrease in Recall and F1 score. There is a compromise on this as most of the incorrect predictions for class A were distributed in other normal case-related morphological patterns. The pathological case-related morphological patterns (LD and FS) and suspect case-related morphological patterns (E and SUSP) had very good performance analysis metrics.

Another aim of this research is to provide ease to the future authors to select the tuned hyperparameters from this work in their work related to cardiotocography with machine learning. This borrowed knowledge would increase the net productivity of any future related work in this field.

A major issue of using this CTG dataset [15] is that this dataset has been derived from subjects of a developed country. Moreover, the sociological, demographic, and medical characteristics (such as maternal nutritional data, maternal health, etc.) of the subjects are not provided in this CTG dataset. All these variables affect the third-trimester events and can potentially be used to fine-tune the proposed model. Further research is needed to verify the actual performance of the proposed model for given subjects from developing countries. Instead of solely relying on the fixed CTG database, future research can be done on direct hardware integration with the proposed mechanism, which would facilitate real-world clinical trials of on-device CTG classification. The accuracy of the proposed mechanism can further be improved by utilizing a combination of more classifiers in future works. In this research, SMOTE synthetically increased the size of the dataset and an improvement in results were achieved. However, if more real entries are added into the CTG dataset [15], then a further improvement can be achieved. Future work for this research can include a larger and real-time CTG dataset. Moreover, future work for this model can include deployment during multiple stages of labor, as inspired by [40].

As this research used the CTG dataset that was sourced from Sisport 2.0, the proposed model can be generalized to work with CTG datasets that have been sourced from the Sisporto programs. The current version of Sisporto 4.0 [41] is also adapted to the 2015 FIGO guidelines for intrapartum fetal monitoring. Related research [42] also highlights the benefits of utilizing computerized CTG (specifically Sisporto) by concluding that Sisporto has many advantages in clinical practice as compared to traditional CTG analysis. Another research paper [43] corroborates the notion that the inclusion of Sisporto in health care results in reductions in the incidence of hypoxic-ischemic encephalopathy (HIE) and cesarean-based deliveries. Hence, in the domain of CTG, Sisporto and the CTG dataset related to it provides a good standard. The CTG dataset is widely used in experiments and research relating to CTG (a fact that is also depicted in Table 10).

The comparison of the results of the proposed model with prior related work is given in Table 10. All of the research work displayed in the table also used the same CTG dataset [15], which was used in this research as well. This was done to highlight the merit of this research by linking it with reputed prior related works and also for providing a better comparison. The defining feature of this research is that it proposed a new model that utilized SMOTE, feature extraction, feature selection, and Bayesian optimization to classify and predict (and hence diagnose) both fetal status as well as CTG morphological patterns. Although there are previous studies that utilize multiple machine learning algorithms for classifying and predicting fetal condition, utilizing multiple machine learning algorithms to achieve this task along with countering the CTG dataset [15] class imbalance issue while utilizing the same CTG dataset (and the model) for classifying and predicting the fetal status as well as the CTG morphological pattern, can be considered a novelty of this research. In terms of clinical applicability, the study (also backed by results in Section 4) suggests that the proposed model has the potential to serve as a decision support tool for managing pregnancies. By accurately diagnosing and classifying fetal conditions and CTG morphological patterns, the model can aid healthcare professionals in making informed decisions and providing appropriate therapeutic interventions when necessary. This clinical applicability implies that the model could be integrated into existing healthcare systems (versions of Sisporto or Sisporto-inspired systems) to support prenatal care and delivery management, potentially leading to improved outcomes. The hypothesis (in Section 1) was substantiated by the results. Thus, this proposed model can be used in tandem with the healthcare system to reduce the adverse fetal outcomes. It can be inferred from the results that the accurate diagnosis and classification of fetal conditions, particularly identifying suspect and pathological cases with a good confidence margin. The proposed model could help in timely intervention and appropriate management of high-risk pregnancies. By providing healthcare professionals with a decision support tool to monitor high-risk pregnancies more effectively, there is potential to detect, diagnose, and address complications or adverse outcomes (for both the fetus and the mother) in a timely manner.

## 6. Conclusions

The practical clinical implication of this research is that remote CTG telemonitoring of fetal well-being can be achieved using this proposed model. This would reduce the need for patients to visit clinics and hospitals in the third trimester. Traditional research work in this domain using the CTG dataset [15] focuses mostly on the three fetal statuses. However, in this research work, classification of the CTG morphological pattern and its relationship with the fetal statuses is in the focus, providing an additive experience to the collective CTG knowledge base. We believe that our methods and our model’s salutary performance will be helpful in guiding and motivating researchers to select our model in their future work related to machine learning-based CTG diagnosis. This knowledge would enhance the net productivity of future work in this field.

## Figures and Tables

**Table 1:** Performance Measurement Metrics.

Metrics	Metric Formula
Precision	$\frac{TP}{TP+FP}$
Recall (Sensitivity)	$\frac{TP}{TP+FN}$
F1-Score	$\frac{2TP}{2TP+FP+FN}$
Accuracy	$\frac{TP+TN}{TP+TN+FP+FN}$
Specificity	$\frac{TN}{TN+FP}$

Note: *TP*, true positives; *TN*, true negatives; *FP*, false positives; *FN*, false negatives.

**Table 2:** CTG Features/Predictors.

Features	Code in Dataset	Description
Fetal Heart Rate (FHR) Baseline	LB	FHR measured in the number of heart beats per min
Accelerations	AC	Number of AC per second
Fetal Movements	FM	Number of FM per second
Uterine Contractions	UC	Number of UC per second
Light Decelerations	DL	Number of DL per second
Severe Decelerations	DS	Number of DS per second
Prolonged Decelerations	DP	Number of DP per second
Abnormal Short-Term Variability	ASTV	Percentage of time with ASTV
Mean Short-Term Variability	MSTV	Mean value of STV
Abnormal Long-Term Variability	ALTV	Percentage of time with ALTV
Mean Long-Term Variability	MLTV	Mean value of LTV
Width of FHR Histogram	Width	-
Minimum of FHR Histogram	MIN	-
Maximum of FHR Histogram	MAX	-
Number of Histogram Peaks	NMAX	-
Number of Histogram Zeros	NZEROS	-
Histogram Mode	Mode	-
Histogram Mean	Mean	-
Histogram Median	Median	-
Histogram Variance	Variance	-
Histogram Tendency	Tendency	Left asymmetric, Symmetric and Right asymmetric

**Table 3:** CTG Morphological Pattern Classes.

Morphological Pattern	Code in Dataset	Description
Calm Sleep	A	-
REM Sleep	B	Rapid Eye Movement
Calm Vigilance	C	-
Active Vigilance	D	-
Shift Pattern	E	Shifts between Class A or SUSP
Accelerative/Decelerative Pattern	AD	Fetus Stress Situation
Decelerative Pattern	DE	Vagal Stimulation
Largely Decelerative Pattern	LD	-
Flat-Sinusoidal Pattern	FS	Pathological State
Suspect Pattern	SUSP	-

**Table 4:** Fetal Condition Assessment by FIGO Guidelines [1].

Fetal Condition	Remarks	FHR
		Assessment
Baseline rate is 110–160 bpm, Variability is 5–25 bpm, No deceleration	Fetus has no hypoxia, and acidosis, so no intervention is needed	Normal
At least one characteristic of a normal case is missing while no pathological case is present	Fetus has a low probability of having hypoxia and acidosis, so constant monitoring is required	Suspect
Baseline rate is <100 bpm, reduced variability for >50 min (or increased variability for >30 min or sinusoidal pattern for >30 min), Decelerations are late or prolonged > 30 min (or 20 min if reduced variability or one prolonged deceleration >5 min)	Fetus has a high probability of hypoxia and acidosis, so immediate intervention is required	Pathologic

**Table 5:** Performance Analysis of the Proposed Model for Fetal Status using Metrics of Table 1.

Fetal Status	Precision	Recall	Specificity	F1-Score
Normal	0.9844 (0.9527)	0.9313 (0.9829)	0.9928 (0.8360)	0.9571 (0.9675)
Suspect	0.9247 (0.8333)	0.9800 (0.6944)	0.9619 (0.9782)	0.9515 (0.7575)
Pathological	0.9907 (0.9183)	0.9861 (0.9000)	0.9950 (0.9917)	0.9884 (0.9090)

Note: The values within parentheses ( ) are for the case when only basic RF was used without the proposed algorithm.

**Table 6:** Confusion Matrix of The Proposed Model for Fetal Status.

			Predicted	
		Normal	Suspect	Pathological
**Actual**	**Normal**	93.1% (98.3%)	6.4% (1.5%)	0.5% (0.2%)
	**Suspect**	1.5% (26.4%)	98.0% (69.4%)	0.5% (4.2%)
	**Pathological**	0.0% (2.0%)	1.4% (8.0%)	98.6% (90.0%)

Note: The values within parentheses ( ) are for the case when only basic RF was used without the proposed algorithm. Also for ease of comparison, the entries in the confusion matrix are depicted as percentages.

**Table 7:** Performance Analysis of the Proposed Model for CTG Morphological Pattern using Metrics of Table 1.

Morphology Pattern	Precision	Recall	Specificity	F1-Score
A	0.9389 (0.8073)	0.8092 (0.9166)	0.9938 (0.9518)	0.8692 (0.8585)
B	0.9565 (0.8758)	0.8859 (0.9241)	0.9953 (0.9505)	0.9198 (0.8993)
C	0.9459 (1.0000)	0.9929 (0.6363)	0.9938 (1.0000)	0.9688 (0.7777)
D	0.9934 (0.8750)	0.9934 (0.7000)	0.9992 (0.9960)	0.9934 (0.7777)
E	0.9281 (1.0000)	0.9872 (0.4583)	0.9907 (1.0000)	0.9567 (0.6285)
AD	0.9302 (0.8658)	0.9160 (0.8875)	0.9931 (0.9756)	0.9230 (0.8765)
DE	0.9225 (0.9473)	1.0000 (0.8307)	0.9916 (0.9935)	0.9597 (0.8852)
LD	1.0000 (1.0000)	0.9870 (1.0000)	1.0000 (1.0000)	0.9934 (1.0000)
FS	0.9520 (0.8823)	0.9720 (0.7894)	0.9946 (0.9961)	0.9619 (0.8333)
SUSP	0.9225 (0.7959)	0.9562 (0.9512)	0.9916 (0.9796)	0.9390 (0.8666)

Note: The values within parentheses ( ) are for the case when only basic RF was used without the proposed algorithm.

**Table 8:** Confusion Matrix of The Proposed Model for CTG Morphological Pattern.

	Predicted									
Actual	A	B	C	D	E	AD	DE	LD	FS	SUSP
**A**	**80.9 (91.7)**	1.3 (5.2)	4.6 (0.0)		5.9 (0.0)	0.7 (0.0)	1.3 (1.0)		2.0 (0.0)	3.3 (2.1)
**B**	4.0 (3.4)	**88.6 (92.4)**	0.7 (0.0)	0.0 (1.4)	2.0 (0.0)	4.7 (2.8)				
**C**	0.0 (36.4)	0.7 (0.0)	**99.3 (63.6)**							
**D**		0.0 (25.0)		**99.3 (70.0)**		0.7 (5.0)				
**E**	0.0 (29.2)	0.0 (8.3)			**98.7 (45.8)**					1.3 (16.7)
**AD**		2.3 (8.8)		0.8 (0.0)		**91.6 (88.8)**	5.3 (2.4)			
**DE**	0.0 (6.2)					0.0 (9.2)	**100 (83.1)**			0.0 (1.5)
**LD**							1.3 (0.0)	**98.7 (100)**		
**FS**	0.0 (5.3)								**97.2 (78.9)**	2.8 (15.8)
**SUSP**	1.5 (0.0)								2.9 (4.9)	**95.6 (95.1)**

Note 1: The values within parentheses ( ) are for the case when only basic RF was used without the proposed algorithm. Note 2: For ease of comparison, the entries in the confusion matrix are depicted as percentages. Note 3: The blank entries in the table equate to a value of 0.0.

**Table 9:** Overview of the Bayesian Optimization Module of the Proposed Model.

Parameter	Fetal Status	CTG Morphological Pattern
Number of estimators (the number of trees in the forest)	214	179
Split criterion	Entropy	Entropy
Maximum number of features to consider for a split at a node (max_features)	$\sqrt{number\;of\;features}$	$\sqrt{number\;of\;features}$
Class weights parameter	Balanced mode ^1^	Balanced mode ^1^

^1^ This mode uses the values of y (target output) to automatically adjust weights inversely proportional to class frequencies in the input data.

**Table 10:** Comparison of this research with related works.

Author(s)	Method	Accuracy
Y. Zhang et al. [32]	SVM with AdaBoost	93%
M. Manikandan et al. [12]	RF with Bagging	96.61%
A. Batra et al. [19]	RF	93.41%
Y. Fei et al. [33]	FCM-ANFIS	96.39%
Z. Cömert et al. [24]	Resilient Backpropagation	93.60%
Z. Hoodbhoy et al. [20]	XGBoost	93%
N.J.A. Kadhim et al. [26]	Naïve Bayes classifier with Firefly algorithm	86.54%
K. Agrawal et al. [21]	DT	93.17%
A. K. Pradhan et al. [22]	RF	93%
M. Ramla et al. [23]	CART	90.12%
This Research	Combination of AE, RFE, BO	96.62%

## Data Availability

The authors used an open access dataset that is available from https://archive.ics.uci.edu/ml/datasets/cardiotocography (accessed on 15 January 2022).

## Abbreviations

The following abbreviations are used in this manuscript:

CTG	Cardiotocography
APGAR	Appearance, Pulse, Grimace, Activity and Respiration
ML	Machine Learning
FE	Feature Extraction
AE	Autoencoder
FS	Feature Selection
RFE	Recursive Feature Elimination
RF	Random Forest
ROC	Receiver Operating Characteristic
AUC	Area Under the ROC Curve
PR	Precision-Recall curve
FIGO	International Federation of Gynecology and Obstetrics
NSP	Normal, Suspect, Pathologic
SMOTE	Synthetic Minority Oversampling Technique
SHAP	SHapley Additive exPlanations
STROBE	STrengthening the Reporting of OBservational studies in Epidemiology
EBI	Encoder Bottleneck Information
EBN	Encoder Bottleneck Neurons
$EB_{C}$	Encoder Bottleneck Coefficient
BO	Bayesian Optimization
WHO	World Health Organization

## Appendix A

### Appendix A.1. Hyperparameter Optimization

Hyperparameter optimization is akin to “tuning of a problem”. This is done by using certain parameters (that can be used to control the learning procedure) in such a way as to find those values that are able to optimally solve the machine learning problem. Optimization is achieved by minimizing a predefined loss or maximizing the accuracy of an objective function in given independent data. The “objective function” takes a tuple of hyperparameters while returning the associated loss. Most commonly, cross-validation is used to estimate this generalization performance. The commonly used search algorithms are: Grid search optimization, Random search optimization, and Bayesian optimization. All three types of optimization searches have their niche applications.

Grid Search Optimization is the simplest method to perform hyperparameter optimization. It simply performs an exhaustive search on user-specified hyperparameters. To better utilize this method, the users must have some preliminary knowledge of the hyperparameters. Otherwise, either the search time will increase or important values of the hyperparameter will be ignored. Moreover, grid search works best if a small search space is available. If the search space is large, then the time to converge for a good set of hyperparameters will increase exponentially [38].

Random Search Optimization can be considered as an upgrade on grid search. It performs a randomized search over the hyperparameter search space. By allowing the process to deplete the predefined budget or until a required accuracy is achieved, the search can be terminated. Even though random search is like grid search, it has certain advantages. For instance, for a case where the hyperparameters are not uniformly distributed, random search may perform better compared to using grid search in the same case. Moreover, the increase in the time allocated to perform random search results in an increase in the possibility of finding the optimal hyperparameters. This rationale is also called the Monte Carlo method [44], which has found popular usage when dealing with large multi-dimensional datasets in deep learning [38]. However, in the case of grid search, better results cannot be warranted by the inordinately longer search time. Random search is more effective than grid search, but it is still a computationally resource-intensive approach.

Bayesian Optimization is based on the Bayesian method [45]. This sequential method pursues the global optimum with the least number of iterations. It seeks a balance between exploration, the process of finalizing the best decision on available information, and exploitation. It is a model that strives to collect more information [38]. Bayesian optimization treats every problem as a black box. A probability surrogate model of objectives is conceived. Then, based on previous iterations’ results, every attempt is made to reach the optimum solution (selected best values of the hyperparameters). The steps in the Bayesian optimization algorithm are as follows:

1. A surrogate model’s prior distribution is made.
2. The best performing hyperparameter set on the surrogate model is obtained.
3. The acquisition function is computed for the current surrogate model.
4. The selected hyperparameter set is applied on the objective function.
5. The surrogate model is updated with the new results.

These steps are repeated until the resource is exhausted or an optimal hyperparameter set is found.

### Appendix A.2. Impact of Bayesian Optimization on Model

The effect of Bayesian optimization (BO) on the proposed model can be observed by comparing the results of the proposed model (with 13 features selected via the RFE module) with BO implementation and results of the proposed model without BO implementation. The performance analysis (using performance metrics given in Table 1) and confusion matrix are given in Table A1 and Table A2, respectively.

**Table A1:** Performance Analysis of the Proposed Model for Fetal Status using Metrics of Table 1: Observing the Impact of BO.

Fetal Status	Precision	Recall	Specificity	F1-Score
Normal	0.9844 (0.9844)	0.9313 (0.9338)	0.9928 (0.9928)	0.9571 (0.9584)
Suspect	0.9247 (0.9205)	0.9800 (0.9825)	0.9619 (0.9595)	0.9515 (0.9505)
Pathological	0.9907 (0.9929)	0.9861 (0.9792)	0.9950 (0.9962)	0.9884 (0.9860)

Note: The values within parentheses ( ) are for the case when no Bayesian Optimization is used in the proposed algorithm.

**Table A2:** Confusion Matrix of The Proposed Model for Fetal Status: Observing the Impact of BO.

		Predicted		
		Normal	Suspect	Pathological
**Actual**	**Normal**	93.1% (93.4%)	6.4% (6.1%)	0.5% (0.5%)
	**Suspect**	1.5% (1.5%)	98.0% (98.3%)	0.5% (0.2%)
	**Pathological**	0.0% (0.0%)	1.4% (2.1%)	98.6% (97.9%)

Note: The values within parentheses ( ) are for the case when no Bayesian Optimization is used in the proposed algorithm. For ease of comparison, the entries in the confusion matrix are depicted as percentages.

Apart from the slight increase (of 0.08%) in model accuracy from 96.54% (without BO) to 96.62% (with BO), the important impact of using BO to the proposed model is that it increased the model classification performance for the pathological cases. The values of some performance metrics remained the same for both normal and suspect cases. However, the main reason to use BO in the proposed model was to improve the predictions of the pathological cases. For instance, the correct prediction of pathological cases increased about 0.71%, and the incorrect prediction of pathological cases as suspect cases decreased about 33.33%. In a sensitive area such as fetal well-being, even these minute improvements can save more lives.

### Appendix A.3. SHAP Analysis

SHAP (SHapley Additive exPlanations) analysis is a method for explaining the results of machine learning models. It aims to demonstrate the impact of different traits or input elements on the prediction power of a model. Calculating the Shapley values, which are based on cooperative game theory, is the primary objective of SHAP analysis [39]. These values can be used quantify the contribution of each attribute to the final result. These numbers provide an approximation of the marginal contribution of each feature to the prediction by averaging over all possible feature permutations. Utilizing SHAP analysis in machine learning can lead to the following benefits:

1. Justify the significance of input features.
2. Alter the influence of features on the model by tweaking the weights.
3. To discover bias.
4. To increase the interpretability of the model.

### Appendix A.4. Balancing the Dataset

A balanced dataset is one in which the distribution of classes in the target output are equal. In contrast, an imbalanced dataset has unequal class distribution. Performing classification on an imbalanced dataset can skew the result in such a manner that the model gets less data elements (entries) for minority classes. Hence, the performance of the model for minority classes can suffer. To counter an imbalanced dataset, methods such as undersampling (the majority class) and oversampling (the minority class) can be used. Undersampling can be easier to implement as it randomly eliminates majority class entries in the dataset, but it is not desirable especially in a medical-centric dataset where each entry is important. Oversampling, on the other hand, is a better option, in which the minority class is increased to match the majority class. Random oversampling can be achieved by randomly duplicating the minority classes multiple times. Another oversampling technique, the so-called Synthetic Minority Oversampling Technique (SMOTE), generates synthetic entries in the dataset by interpolating minority class entries into the feature space of data. SMOTE achieves this by creating new instances between a minority sample and its k neighbors. The new entries are not copies of existing entries, but rather their features have a combined effect from the surrounding neighbors. SMOTE only affects the minority class distribution of an imbalanced dataset. It does not affect the majority class. A simplified algorithm [35] of SMOTE is as follows:

1. In a minority class set *M*, such that each x∈M, by calculating the Euclidean distance between *x* and each entity in set *M*, the *k* nearest neighbors of *x* are obtained.
2. The imbalanced ratio is used to set the sampling rate *R*. For each x∈M, *R* samples (x1,x2,…xr) are randomly selected from its *k* nearest neighbors, and they construct the set M1.
3. For each sample xk∈M1 (where k=1,2,3…R), a new entry is generated via: $x^{\prime }=x+rand\left(0,1\right)*|x-xk|$.
